# The brain’s sense of walking: a study on the intertwine between locomotor imagery and internal locomotor models in healthy adults, typically developing children and children with cerebral palsy

**DOI:** 10.3389/fnhum.2014.00859

**Published:** 2014-10-27

**Authors:** Marco Iosa, Loredana Zoccolillo, Michela Montesi, Daniela Morelli, Stefano Paolucci, Augusto Fusco

**Affiliations:** ^1^Clinical Laboratory of Experimental Neurorehabilitation, IRCCS Fondazione Santa LuciaRome, Italy; ^2^Department of Children Neurorehabilitation, IRCCS Fondazione Santa LuciaRome, Italy; ^3^School of Physiotherapy, University of Rome Tor Vergata, IRCCS Fondazione Santa LuciaRome, Italy

**Keywords:** gait, motor imagery, internal model, locomotion, locomotor body schema, cerebral palsy

## Abstract

Motor imagery and internal motor models have been deeply investigated in literature. It is well known that the development of motor imagery occurs during adolescence and it is limited in people affected by cerebral palsy. However, the roles of motor imagery and internal models in locomotion as well as their intertwine received poor attention. In this study we compared the performances of healthy adults (*n* = 8, 28.1 ± 5.1 years old), children with typical development (*n* = 8, 8.1 ± 3.8 years old) and children with cerebral palsy (CCP) (*n* = 12, 7.5 ± 2.9 years old), measured by an optoelectronic system and a trunk-mounted wireless inertial magnetic unit, during three different tasks. Subjects were asked to achieve a target located at 2 or 3 m in front of them simulating their walking by stepping in place, or actually walking blindfolded or normally walking with open eyes. Adults performed a not significantly different number of steps (*p* = 0.761) spending not significantly different time between tasks (*p* = 0.156). Children with typical development showed task-dependent differences both in terms of number of steps (*p* = 0.046) and movement time (*p* = 0.002). However, their performance in simulated and blindfolded walking (BW) were strictly correlated (*R* = 0.871 for steps, *R* = 0.673 for time). Further, their error in BW was in mean only of −2.2% of distance. Also CCP showed significant differences in number of steps (*p* = 0.022) and time (*p* < 0.001), but neither their number of steps nor their movement time recorded during simulated walking (SW) were found correlated with those of blindfolded and normal walking (NW). Adults used a unique strategy among different tasks. Children with typical development seemed to be less reliable on their motor predictions, using a task-dependent strategy probably more reliable on sensorial feedback. CCP showed less efficient performances, especially in SW, suggesting an altered locomotor imagery.

## Introduction

“Go where I’m looking, not look where I’m going”, with this expression Alain Berthoz in his book “The Brain’s sense of movement” claimed the role of gaze-based feed-forward control involved in locomotion along a desired trajectory (Berthoz, 2000). In fact, gaze turns towards the desired trajectory in advance of the feet, suggesting that subjects follow an internal model of the predicted trajectory. When visual feedback is not available, such as during walking in a dark environment, control of locomotion should rely even more on motor predictions (Iosa et al., 2012a).

Two neural mechanisms underlie the mental representation of an action (Ito, 2008; Wolpert and Flanagan, 2010): motor imagery (Beisteiner et al., 1995) and internal motor models (Kawato, 1999). Motor imagery has been defined as a voluntary dynamic state during which a subject mentally simulates a given action (Decety, 1996). Similar neural structures are involved in motor imagery and movement execution: parietal cortex, cortical motor areas, basal ganglia and cerebellum (Decety, 1996; Jeannerod, 2001). Many studies reported that the time imagined as needed for executing an action is strictly related to that really needed to complete that action. Motor imagery is developed during childhood and it reaches an asymptote during adolescence (Smits-Engelsman and Wilson, 2013). This development has been found altered in children with cerebral palsy (CCP; Mutsaarts et al., 2006, 2007).

An internal model is an implicit cerebellar neural mechanism (Wolpert et al., 1998), that can mimic the input/output characteristics, or their inverse, of real structures of our body (such as sensori-motor apparatus (Kawato, 1999)) or of the external environment (such as ecological invariants (McIntyre et al., 2001; Zago et al., 2004)). Internal models are conceivably located into the cerebellum and allow for anticipating consequences of an action given the actual state and a-priori information (feedforward internal models) or for computing the commands needed to obtain a desired trajectory (inverse internal models) (Imamizu et al., 2000).

It has been suggested that motor imagery can be regarded as the conscious experience of internal models (Jeannerod, 2001). On the other hand, internal sensori-motor models could be useful both during motor performances in predicting the motion of body segments as well as during motor imagery (Gentili et al., 2004). The development of motor imagery during childhood was hypothesized to be related to the fact that with age, children become less reliant on feedback and more attuned to feedforward control of movements, capturing aspects of motor prediction and involving a-priori information in their motor behaviors (Smits-Engelsman and Wilson, 2013).

Many studies were focused on the mental representation of walking, mainly based on two different approaches: those in which subjects were explicitly asked to imagine to walk and mental chronometry paradigm was used to verify if imagination time is related to real movement time (Decety, 1996; Bakker et al., 2007), and those in which subjects were asked to perform an action without the support of feedback, such as blindfolded walking (BW) towards a target the position of which was previously memorized (Dominici et al., 2009; Iosa et al., 2012a). This last ability implies the use of an internal model, sometimes called locomotor body schema for computing the needed number of steps suitable to achieve the target in absence of visual support (Dominici et al., 2009; Ivanenko et al., 2011). Despite locomotor imagery and locomotor internal model are conceivably intertwined (Jeannerod, 2001; Gentili et al., 2004), subjects’ performances during walking imagery task and walking distance estimation have been rarely compared (Decety et al., 1989).

Many studies reported an alteration of motor imagery in CCP (Mutsaarts et al., 2006, 2007; Crajé et al., 2010), despite a recent study questioned it for locomotor imagery (Spruijt et al., 2013). In children with developmental coordination disorder their problems in generating a mental representation of an intended action have been hypothesized to be associated with internal modeling deficit (Gabbard and Bobbio, 2011). No studies investigated if the alterations in motor imagery in CCP have been due to (or associated with) alterations in their locomotor internal model, evaluated by means of a walking distance estimation test performed without visual support.

According with the scenario depicted above, we tested locomotor imagery and locomotor internal model in three populations of subjects hypothesizing that their intertwine could be: (1) strict in healthy adults, (2) not completely formed yet in typically developing children (TDC); and (3) altered in CCP.

## Material and methods

### Participants

Three groups of subjects were enrolled in this study: healthy adult group (HAG: 28.1 ± 5.1 years, age range: 23–37 years; 8 subjects: 3 males and 5 females), children clinically defined as typically developing by their pediatrician (TDC: 8.1 ± 3.8 years, age range: 4–14 years; 8 subjects: 4 males and 4 females) and a group formed by (CCP: 7.5 ± 2.9 years, 12 subjects: 7 males and 5 females). The two groups of children were age-matched (*p* = 0.785, u-test). CCP were affected by hemi- or diparesis, had a walking ability classified as level I in the Gross Motor Function Classification System (Palisano et al., 1997), and were able to understand the given instructions (IQ >= 49 assessed by a psychologist using the revised version of Wechsler Intelligence Scale for Children). Their age range was 4–12 years; their mean IQ was 85 ± 20 (range: 53–110). Four subjects had left hemiparesis, five right hemiparesis, three subjects had diparesis.

Local ethics committee approved the study procedures, designed in accordance to the Declaration of Helsinki on human experimentation and the signed informed consent of adult subjects or parents/legal guardian of children were obtained.

### Tasks

Because mental representation by itself is difficult to be measured, all previous studies used paradigms in which outcome measures were hypothesized to be representative of these internal representation, such as measuring response time in Hand Laterality Judgment Task to assess motor imagery (Boonstra et al., 2012). In our study, subjects performed the three tasks described in details below: simulated walking (SW) to assess locomotor imagery, BW to assess locomotor internal model, and normal walking (NW).

Subjects were asked to stand on a strip of tape fixed on the ground (starting line) and to image or to actually walk towards one of two possible targets formed by two other strips placed on the ground at 2 and 3 m from the starting-line and parallel to that. All subjects performed the tasks wearing their common shoes. For each one of the following tasks, both distances (2 and 3 m) were tested in a randomized order. Because it has been demonstrated a practice effect occurring in repeated measurements of motor imagery tasks, implying a progressive improvement of the performances over trial repetition (Philbeck et al., 2008; Boonstra et al., 2012), only one trial was performed for each distance. So, instead of testing two or more trials for the same distance, we preferred to test two slightly different distances for avoiding the phenomenon of learning and/or progressive recalibration, similarly to what done in some previous studies (Smith et al., 2010; Iosa et al., 2012a). Only two distances were tested for avoiding the possible reduction of compliance in children. Target distances were set at 2 and 3 m because it has been shown that even healthy adults significantly undershot the target in indoor environments for distance longer than 3 m, probably because adopting a conservative strategy leaded by the fear to hit a wall (Iosa et al., 2012a).

In the first task SW, subjects were asked to image to walk towards the target and at the same time to simulate walking by means of stepping in place. No instructions were given about looking or not the target during imagination, so subjects could freely decide where to look or to close their eyes. No information was given about the fact that we recorded their number of steps and movement time, neither these parameters were mentioned to subjects.

In the second task BW subjects were asked to walk towards the target after being blindfolded. During these trials, subjects were reassured that an experimenter can promptly advise them if they were going to hit a wall, but none of them needed his intervention. According to previous study (Iosa et al., 2012a), to avoid some possible learning effect of the first trial on the second one, no verbal feedback was given to the participants about their performances and they had been guided back to the starting-line still blindfolded by the experimenter.

In the last task NW, subjects were asked to stand on the starting-line and then to achieve by NW the target line formed by tape on the ground with their eyes opened at their self-selected comfortable speed. This test was performed in order to measure the normal self-selected spatio-temporal gait parameters under visual control.

### Measures

All the tests were performed within a rectangle (length: 6 m, width: 2.5 m) formed by optoelectronic bars placed on the ground in our laboratory (Optogait^®^ with inertial unit gyko, Microgate, Italy; sampling frequency = 100 Hz). Half of the electronic bars contained an infrared light emitter each 1.04 cm and the other half a receiver at the same distance. This optoelectronic system was used for measuring the number of performed steps and their spatio-temporal related parameters. In the blindfolded task, analogously to previous studies (Iosa et al., 2012a,b), the experimenter also measured the distance between the target and the middle point of the two malleoli of the subject with a graduated tape, increasing the resolution from 1 cm to 1 mm. During all the above tasks, participants wore an elastic belt containing a wireless triaxial accelerometer (inertial unit gyko, sampling frequency = 100 Hz) located on the back in correspondence of L2–L3 spinous processes, close to the subject’s center of mass, and providing acceleration signals along the three body axis.

The outcome measures for all the three tasks were: number of performed steps (measured by optoelectronic system) and movement time (measured by accelerometer). SW performance was hypothesized to be informative on motor imagery, BW performance on locomotor internal model, and NW was used as reference condition. The accelerometer allowed for identifying start and stop of subject’s movements (as done in previous studies (Iosa et al., 2012a,c,d)) and their time difference represented the movement time, i.e., the time spent by subjects during simulated and actual walking. For BW also the error (as well as its absolute value independent by under- or over-shooting the target) was measured as the difference between walked distance and real target distance and it was expressed in percentage of real target distance.

### Statistical analysis

Mean and standard deviation were computed for summarizing the outcome measures. Because of the small sample sizes and because not all the data sets resulted normally distributed, non parametric statistics was used for analyzing data. Friedman’s analysis was performed to assess the effect of task (3 levels: simulated, blindfolded, and NW) within groups. For HCG and TDC the number of data included into that analysis was 16 (8 subjects for each group per two distances), whereas for CCP it was 24 (12 subjects for 2 distances). *Post-hoc* comparisons were performed using Wilcoxon signed rank test.

The correlation between the values of each parameter recorded in two different tasks was computed on the above data sets using Spearman’s coefficient (R). Percentage differences with respect to NW were computed as the difference between values recorded in SW or BW and that of NW, divided by NW-value and multiplied per 100. Percentage walking error in blindfolded task was computed as distance from target divided by target distance and multiplied per 100. Comparisons among groups were performed using Kruskal-Wallis analysis, and Mann-Whitney u-test was applied for comparing two independent samples, such as in between group *post-hoc* analyses.

The threshold for statistically significant difference was set at 0.05 for all the analyses, but for *post-hoc* tests for which Bonferroni correction was applied.

## Results

**Figure 1:**
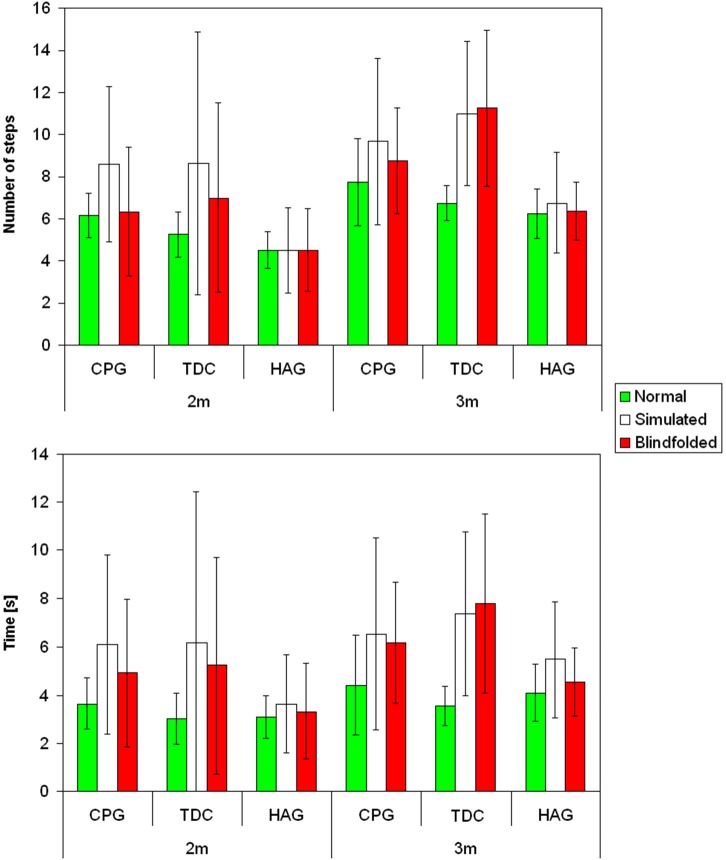
**Mean and standard deviations for the number of steps (above) and movement time (below) for children with cerebral palsy (CCP), typically developing children (TDC) and healthy children group (HCG) during normal (green), simulated (white) and blindfolded (red) walking for the two tested distances**.

Figure 1 shows mean and standard deviation of number of steps and movement time for the three groups in the three tasks for the two tested distances. Table 1 shows the results of Friedman’s analyses and relevant *post-hoc* comparisons.

**Table 1:** **Within group comparisons of number of steps and movement time in healthy adults (HAG), typically developing children (TDC) and children with cerebral palsy (CCP)**.

Parameter	Group	Friedman’s analysis		*Post-hoc* analyses Wilcoxon Signed Rank Test (*p*-value)		
		χ^2^	p	SW vs. NW	BW vs. NW	SW vs. BW
Number of steps	HAG	0.545	0.761	—	—	—
	TDC	0.614	**0.046**	**0.010**	**0.006**	0.475
	CCP	7.624	**0.022**	**0.015**	0.175	0.068
Movement time	HAG	3.714	0.156	—	—	—
	TDC	12.133	**0.002**	**0.004**	**<0.001**	0.798
	CCP	24.343	**<0.001**	**<0.001**	**<0.001**	0.170

### Healthy adult group (HAG)

In healthy adults, neither the number of steps nor the movement time significantly differed between tasks for healthy adults (Table 1). The percentage differences with respect to NW were lower for number of steps than for movement time in SW (4% vs. 26%, respectively, *p* = 0.011) and slightly in BW (1% vs. 10%, *p* = 0.100).

Significant correlations were also found between the recorded values of each parameter in different tasks (Table 2). The only exception was the correlation of movement time values between SW and BW, that only approached the significant threshold (*p* = 0.089).

**Table 2:** **Spearman’s correlation coefficient (***R***) between two conditions for time and number of steps**.

	Movement time	Simulated	Blindfolded	Normal
	Number of steps	walking	walking	walking
	Simulated	—	0.439	0.616*
HAG	Blindfolded	0.743**	—	0.629**
	Normal	0.547*	0.738**	—
	Simulated	—	0.673**	0.059
TDC	Blindfolded	0.871**	—	−0.012
	Normal	0.504*	0.682**	—
	Simulated	—	0.344	0.690**
CCP	Blindfolded	0.034	—	0.572**
	Normal	−0.070	0.669**	—

*p <= 0.05, **p <= 0.001.

Some of adult subjects undershot the target during BW, resulting in a negative mean spatial error (−13 ± 11%, in percentage of the distance). The corresponding mean absolute error was 13 ± 11%.

### Typically developing children (TDG)

As observable in Figure 1, for children with typical development, the performances of simulated and BW were similar each other, but quite different from that of NW. In fact, both number of performed steps and especially movement time significantly differed between tasks for these children (Table 1). It was due to a significantly higher number of steps and longer time spent during both blindfolded and simulated conditions with respect to NW.

The percentage differences with respect to NW were higher than those observed for healthy adults, but remained lower for number of steps than for movement time (SW: 60% vs. 112%, *p* = 0.017; BW: 49% vs. 97%, *p* = 0.010, respectively).

Analogously, significant correlations were found between tasks for the number of steps, whereas the movement time resulted significantly correlated only between SW and BW (Table 2).

During BW, the error of children was in mean close to zero: −2.2 ± 8.9%, with an absolute error of 12.3 ± 7.3%, similar to that found for HAG.

### Children with cerebral palsy (CCP)

Figure 1 shows that, the performances of CCP were partially similar to those of children with typical development, but some peculiar differences. Similarly to them (and differently from healthy adults), number of steps and movement time resulted both dependent on tasks (Table 1). *Post-hoc* analyses revealed that, especially for number of steps, it was mainly due to differences between simulated and NW. Similarly to HAG and TDC, the percentage differences of number of steps with respect to NW were lower than the related differences of movement time (SW: 37% vs. 47%, *p* = 0.458; BW: 8% vs. 39%, *p* < 0.001).

**Figure 2:**
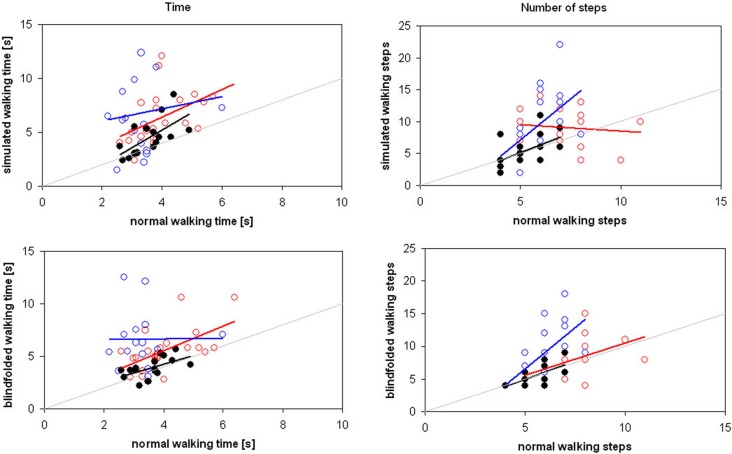
**Time (on the left) and number of steps (on the right) spent for covering the distances of 2 and 3 m in simulated (above) and blindfolded (below) walking by adults (black filled circles), children with typical development (blue empty circles) and children with cerebral palsy (red empty circles)**. Regression lines are reported in the same color of relevant data, whereas the gray line is the theoretical line of equality.

**Figure 3:**
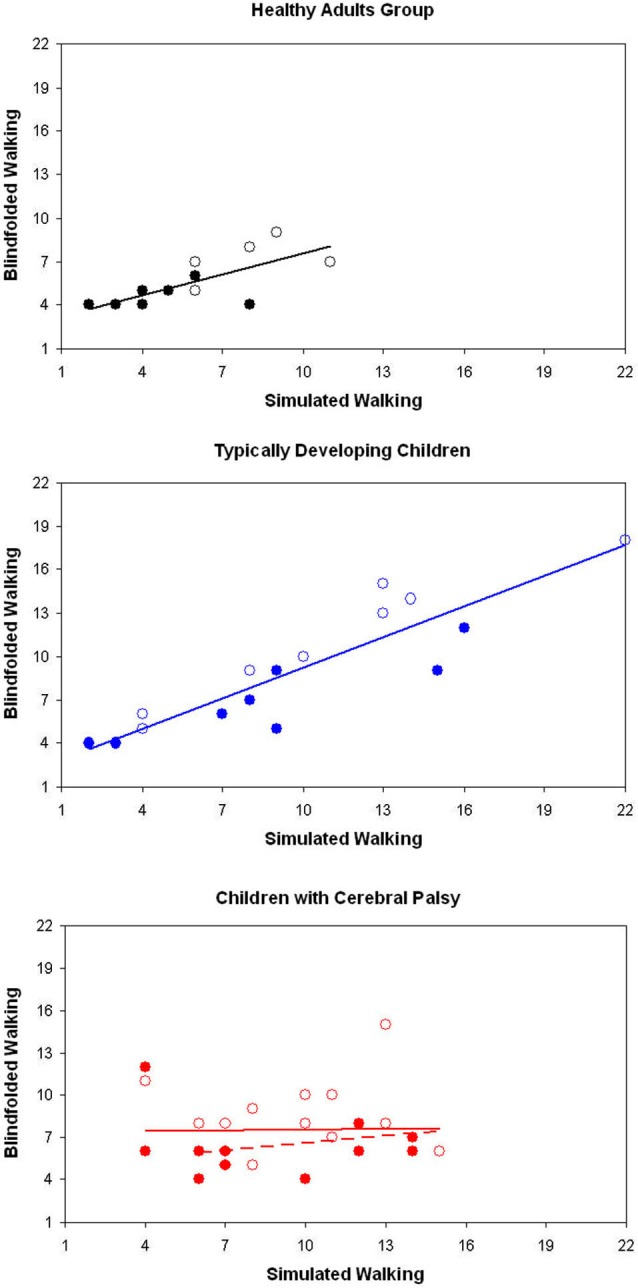
**Number of steps recorded during simulated and blindfolded walking for healthy adults (above), typically developing children (in the middle) and children with cerebral palsy (CCP), for the distance of 2 m (filled markers) and 3 m (empty markers)**. Regression lines are reported in the same color of all the relevant data.

Differently from children with typical development, the number of steps of CCP during SW was not significantly correlated neither with that of NW nor with that of BW (Table 2, Figure 2). This result was independent by the target distance (Figure 3). It was partially due to six out of the 12 children with CP, who did not imagine that more steps were needed for covering 3 m with respect to 2 m during SW. None adult and just one child with TD showed a similar behavior. Because during BW, this rate was reduced to 2 out of 12 children with CP, the correlation between the number of steps performed in BW and SW was not statistically significant. Moreover, this correlation was completely absent in these six children (*R* = −0.114, *p* = 0.724). If these subjects were excluded from the analysis, the correlation slightly improved, but not significantly (*R* = 0.310, *p* = 0.326). Neither age (7.0 ± 3.2 vs. 8.0 ± 2.6, *p* = 0.375) nor IQ (93.2 ± 20.3 vs. 80.3 ± 16.2, *p* = 0.093) resulted significantly lower in these children with respect to the other six children. Neither the number of steps really performed by these two subgroups of children with CP resulted statistically different (for 2 m: *p* = 0.699, for 3 m: *p* = 0.280). Of these six children, three had left hemiparesis, two right hemiparesis, and one diplegia (the other group was formed by three children with right hemiparesis, two with diplegia and a child with left hemiparesis).

The correlation between number of steps in SW and BW remained not statistically significant even when re-evaluated using as covariate IQ-score, age or number of steps needing during NW (IQ: *R* = 0.047; age: *R* = 0.136, steps: *R* = 0.095, respectively, *p* > 0.5 for all of them).

The mean error performed during BW was −15.5 ± 21.2% for CCP, with an absolute error of 19.6 ± 17.3%. The mean error was significantly different in the three groups of subjects (χ^2^ = 9.281, *p* = 0.010), and *post-hoc* analyses revealed that this difference was significant between CCP and TDC (*p* = 0.008), but not between CCP and HAG (*p* = 0.658).

## Discussion

### Key findings

The aim of this study was to investigate the intertwine between locomotor imagery and internal locomotor model in three groups of subjects. Our results confirmed the hypothesis of a strict intertwine in healthy adults, who showed similar performances in all the three tasks. As shown in Figure 3, their performances were correlated also between simulated and BW.

We also hypothesized that this intertwined was not completely formed in TDC. We found that their performance was task-dependent, despite low errors, suggesting an adaptable motor behavior. Anyway, their performances between simulated and BW were strictly correlated. Finally, we hypothesized that the intertwine between locomotor imagery and locomotor internal model could be loss in CCP. Our results confirmed it, suggesting a more marked deficit in motor imagery than in internal model.

The performances in terms of movement time were found even more statistically different between tasks for the two groups of children, and with larger differences with respect to NW in all the three groups. It suggested that the parameter leading the motor strategies was the number of steps. Healthy adults performed in simulating and blindfolded tasks about the same number of steps performed during NW, showing a good ability in estimating the actual number of steps needed for traveling a given distance. It is in accordance with the suggestion that subjects encoded a seen distance in terms of “action units” more than in terms of meters: for adults involved in our study these action units seemed to be the steps needed for covering that distance (Berthoz, 2000). These results can also be read in conjunction with those of Dominici et al. (2009) about blindfolded healthy subjects walking on stilts who undershot the target. Authors explained these results suggesting that subjects had probably planned a reduced number of “expected” longer strides with their lengthened legs, but in real, their actual step length was not longer for a reduction of hip sagittal range of motion, resulting into a reduced traveled distance. This behavior implied that subjects did not take into account proprioceptive signals related to the reduction of hip flexion/extension range or otolith signals related to head forward movement, but based their strategy only on the prediction of the needed steps. It is conceivable that also in our study healthy adults estimated the number of needed steps independently by the task.

Children with typical development showed a different strategy, adapting their number of steps in a task-dependent manner. Despite the differences, the correlation between simulated and BW was statistically significant for both number of steps and movement time. The correlation was statistically significant also for number of steps in simulated and BW with respect to NW, but not for movement time.

These results are in accordance with those reporting that correlation between real and imagined movement times is poor under 8 years, increases during adolescence, and is robust only in adults (Skoura et al., 2009; Smits-Engelsman and Wilson, 2013). Despite it, TDC showed good performances in achieving the target during BW. They adapted their walking in absence of visual feedback performing a higher number of steps and spending more time to complete the trial, probably also taking into account their sensorial feedback. Children could be less self-confident of adults in their internal representations and rely on sensorial feedbacks for selecting their locomotor strategy (Smits-Engelsman and Wilson, 2013). The reason for which children are less reliant in prediction of movements could be related to the fact that they had not yet a standardized comfortable step length. In fact, they are still developing, implying a progressive increase of step length, that is not only proportional to anthropometric growth (Sutherland et al., 1980), but also related to changes in thigh, shank and foot kinematics (Ivanenko et al., 2004). Also brain development should be taken into account. It has also been shown that the relationship between motor imagery and motor skill becomes stronger with age (Caeyenberghs et al., 2009), achieving an asymptote after adolescence (Smits-Engelsman and Wilson, 2013). Hence, motor imagery development has been suggested to follow brain development and to reflect the unfolding of internal modeling processes in healthy subjects (Caeyenberghs et al., 2009).

However, we found differences between imaging (simulating) walking and predicting the effect of their (blindfolded) walking (i.e., the achievement or not of the target) in CCP. It conceivably suggests that motor imagery ability and its development can only captures some aspects of the implicit processes involved into motor prediction (Smits-Engelsman and Wilson, 2013), and this cognitive capture could be difficult for children with CP. In fact, in CCP, neither number of steps nor movement time resulted significantly correlated between simulated and blindfolded performances. It was mainly due to altered locomotor imagery. In fact, differently from other subjects and differently from real walking, half of children with CP did not imagine more steps needed for covering the longer distance with respect to the shorter one. These subgroup of children were neither younger nor with lower IQ than the other children with CP. Anyway, also in the other children the correlation between simulated and blindfolded performances was poor.

During BW, their mean errors remained quite higher than those performed by age-matched TDC. BW could be a difficult task for children with CP also for their reduced upright gait stability (Iosa et al., 2012d, 2014). However, both number of steps and movement time were found correlated between blindfolded and NW. These results suggested that their motor imagery could be more markedly impaired than their locomotor internal model.

There are some factors that could have affected the performance of CCP during SW, mainly their motor and their cognitive impairments. Further, age could influence both cognitive and motor performances. However, correlation between simulated and BW remained not statistically significant even when locomotor functioning, IQ or age were introduced as covariates. It could be due to the reduced sample size, as reported into the next session about the limit of our study. Howsoever, it was evident that the performance of children with CP was more altered during simulated than during BW. It could be possible that these children needed to have sensorial feedback to improve their performances. Further, their internal locomotor model, being located into cerebellum, could be less impaired than their motor imagery, involving brain areas potentially affected by cerebral palsy.

### Limits

The main limit of our study was the reduced size of enrolled samples. Despite in line with previous studies (Mittelstaedt and Mittelstaedt, 2001; Stevens, 2005), and sufficient to highlight statistically significant differences, the reduced sample size did not allow us for dividing subjects in homogenous subgroups, for example for dividing children with CP in relationship to the damaged brain areas. Further studies should clarify how the effects of damages in specific areas could impair motor imagination and locomotor predictions.

Then, SW tasks in this study can not be considered as a pure protocol of motor imagery, because it involved stepping in place for eliciting information about motor imagery. Further, subjects involved in this study were asked to perform a “spatial” task towards a target, without any kind of time constraints: it could have limited the role played by time estimations in subjects’ performances. The fact that the distances were the same for all the subjects could have increased the task difficulty for CCP because they needed a higher number of steps for covering the given distances. However, their performance in imaging to walk towards a target located at 2 m from them remained poorer than that of TDC for the distance of 3 m. Finally, for avoiding learning effects, subjects performed each trial just one time, not allowing for averaging performances on a wide amount of trials.

### Theory and future researches

It is not so common for a healthy adult to walk without visual feedback, despite it sometimes happens, especially during night in his/her own house. This ability implies the construction of a mental map of the surrounding environment, but it is also related to the ability of imaging ourselves moving on that map (Palermo et al., 2008). What is the unit of measure of that neural map? Even if adult subjects have poor performance in judging a distance in terms of meters (Iosa et al., 2012a), our results showed that subjects were able to estimate the correct number of steps needed to achieve a target. So, it suggests the hypothesis of an inverse internal model in which a standard step length is encoded as action unit for estimating distances in terms of number of steps needed to cover them.

Children with typical development showed a task-dependent strategy: their more adaptable motor behavior seemed to be less reliant on a-priori internal predictions. However also their performances resulted accurate and it could be due to a proper exploitation of sensorial afferent feedback. How can sensorial feedback be transformed into information about traveled distance? Even if including sensorial feedback this processing needs an internal model. The first possibility is that subjects can use an internal model exploiting their proprioceptive signals of lower limb joint angles and a-priori information about their lower limb length (internalized into their body schema) (Mittelstaedt and Mittelstaedt, 2001; Dominici et al., 2009). Alternatively, traveled distance could be estimated by double-integration of the otolith signals, i.e., by an internal model doubly integrating head accelerations. This last hypothesis is supported by accurate performances of subjects even during blindfolded passive target-directed translations (Israel et al., 1997). It has also been suggested that inertial and proprioceptive information could be combined and optimized in a task-dependent weighted averaging (Mittelstaedt and Mittelstaedt, 2001). Our results can not clarify which one of these models, or if a combination of them was used by TDC and further studies are needed.

In accordance with literature (Mutsaarts et al., 2006, 2007), CCP showed poor capacity of imagining their motor actions. Further, the errors performed by these children in BW were systematic and brought them to undershoot the target. Many explanations are possible and need further studies to be deeply investigated: a reduced upright gait stability that may lead them to use a more conservative strategy (Iosa et al., 2012a), an altered proprioception (Riquelme and Montoya, 2010), an altered body image (Hammar et al., 2009), or an altered internal locomotor model. This last hypothesis opens a scenario that needs further studies and related to the action-observation processing in children with an altered development. They usually observe healthy subjects, for example their typically developing age-matched school mates, walking with longer steps, and it could contribute to form an altered locomotor body schema, overestimating their walking ability. It has been shown that the subliminal activations of motor pathways during observation of others’ actions, mediated by the mirror neuron system, were different between possible and impossible actions, even if these actions have an identical intention (Borroni et al., 2011). What happens when a subject for whom an action is impossible observes another subject performing that action? However, it is noteworthy that even if the performances of CCP during BW showed an error resulting in undershooting the target, the number of steps was found correlated with NW. It did not happen for simulating walking, suggesting the possibility that motor imagery could be more affected than internal locomotor models in these children. Further studies are needed to investigate this scenario, especially because new action-observation rehabilitative protocols have recently been proposed for these children.

Although only a small part of human motor activity is reflected at the conscious level, motor and sensory components of action are deeply intertwined, suggesting inherent linkage between perception and action in the system of internal representation (Jeannerod, 2001; Rizzolatti and Sinigaglia, 2007; Ivanenko et al., 2011). Our results supported the hypothesis of the development of a fascinating link between the internal models probably stored in the cerebellum and their conscious cerebral counterpart forming a sense of walking into the brain.

## Conflict of interest statement

The authors declare that the research was conducted in the absence of any commercial or financial relationships that could be construed as a potential conflict of interest.
